# Attenuated Molecular Response to SARS-CoV-2 in MDMs Isolated from Immunosuppressed Transplanted Patients

**DOI:** 10.3390/ijms262110489

**Published:** 2025-10-28

**Authors:** Roberta Vazzana, Josè Camilla Sammartino, Nicola Cuscino, Roberto Giambruno, Claudia Carcione, Vitale Miceli, Matteo Bulati, Valentina Agnese, Daniele Lilleri, Pier Giulio Conaldi, Fausto Baldanti, Irene Cassaniti, Alessia Gallo

**Affiliations:** 1 Department of Research, IRCCS-ISMETT (Istituto Mediterraneo per i Trapianti e Terapie ad Alta Specializzazione), 90127 Palermo, Italy; rvazzana@ismett.edu (R.V.); ncuscino@ismett.edu (N.C.); vmiceli@ismett.edu (V.M.); vagnese@ismett.edu (V.A.); pgconaldi@ismett.edu (P.G.C.); 2 Department of Clinical-Surgical, Diagnostic and Pediatric Sciences, Università Degli Studi di Pavia, 27100 Pavia, Italy; j.sammartino@smatteo.pv.it (J.C.S.); fausto.baldanti@unipv.it (F.B.); i.cassaniti@smatteo.pv.it (I.C.); 3 Institute for Biomedical Research and Innovation, National Research Council, 90146 Palermo, Italy; roberto.giambruno@cnr.it; 4 Fondazione Ri.MED, 90133 Palermo, Italy; ccarcione@fondazionerimed.com; 5 Microbiology and Virology Department, Fondazione Istituto di Ricovero e Cura a Carattere Scientifico (IRCCS) Policlinico San Matteo, 27100 Pavia, Italy; d.lilleri@smatteo.pv.it

**Keywords:** SARS-CoV-2 VOCs, macrophages, RNA-Seq, immunosuppression, immune response, gene expression

## Abstract

Immunosuppressive therapies used in clinics to reduce the risk of rejection in transplanted patients unfortunately also decrease the response of the immune system to the pathogens. Previous data has shown that the most diffuse SARS-CoV-2 variants of concern between 2020 and 2021 showed a different modulation of the host immune response in healthy subjects, with the Delta B.1.617.2 variant leading to a failure in the activation of the adaptive immune response. In this study, the transcriptomic profiles of monocyte-derived macrophages (MDM), isolated from four immunosuppressed kidney transplant patients and exposed to SARS-CoV-2 VOCs, were analyzed and compared with previously published data gathered from immune-competent subjects. Human monocytes were isolated from peripheral blood mononuclear cells (PBMCs) of four kidney transplant patients admitted to the IRCCS Policlinico San Matteo of Pavia (Italy), differentiated into macrophages, and exposed to the active and the UV-inactivated particles of the different SARS-CoV-2 VOCs (D614G, Alpha B.1.1.7, Gamma P.1, Delta B.1.617.2 and Omicron BA.1). Bulk RNA-Seq was performed and significant transcripts were assessed based on Student’s *t*-test (*p*-value < 0.05) and Fold change > 2. RNA-Seq data analyses of immunosuppressed MDMs showed that SARS-CoV-2 VOCs, although transcriptionally active, did not induce strong alterations in the transcriptomic profiles of these cells, while a strong down-regulation of key genes involved in the innate immunity pathways was observed when comparing these data to the ones obtained from immunocompetent participants. Overall, this study suggests that patients under immunosuppressive therapies do have an altered macrophage response to SARS-CoV-2 viral infection.

## 1. Introduction

In immunocompetent individuals, who are generally able to accomplish viral clearance, the response to SARS-CoV-2 infection is characterized by a synergy between innate and adaptive immune system reactions. The innate immune system acts in limiting viral entry and replication by activating specific inflammatory pathways and contributes to the activation of the adaptive immune response [1].

Among the innate immunity players, macrophages act as a first line of defense during infections, including SARS-CoV-2 viral infection [2]. The effects of the active and the UV-inactivated SARS-CoV-2 VOCs on the transcriptomic profiles of MDMs isolated from healthy subjects were recently characterized [3,4]. Those studies provided insights on the MDM molecular pathways implicated in the immune evasion that confer the ability to evade a primed immune response, especially for Delta and Omicron variants.

In immunocompromised individuals, including solid organ transplant recipients, susceptibility to viral infections is exacerbated not only by impaired adaptive immunity but also by dysfunctional innate immune responses [5]. Several studies have reported that these patients exhibit altered monocyte and macrophage phenotypes, reduced cytokine production, and attenuated antigen presentation capacity. For instance, a reduced interferon signaling and altered Toll-like receptor responses have been documented in monocyte-derived macrophages (MDMs) from transplant patients, suggesting a compromised first-line defense against viral pathogens [6]. Beyond SARS-CoV-2, this impaired antiviral immunity has also been observed in the context of both cytomegalovirus (CMV) and Epstein–Barr virus (EBV) reactivation, which are highly frequent in transplant recipients under chronic immunosuppressive therapy [7,8]. These findings highlight the need to better characterize how commonly used immunosuppressive drugs can modulate macrophage responses, particularly when these cells are challenged by highly pathogenic respiratory viruses, such as SARS-CoV-2.

During the COVID-19 pandemic, immunocompromised patients showed an increased risk of developing severe symptoms associated with the disease, alongside higher rates of hospitalization and mortality [9]. Previous findings showed that immunosuppressed subjects, such as transplanted patients, are less protected by vaccines compared to the general population and show a higher risk of prolonged infection with SARS-CoV-2 [10].

Considering the crucial role of macrophages in the host defense against viral infections and the transcriptomic alterations observed in human MDMs when exposed to the different VOCs, we wondered whether, under an immunosuppressive therapeutic regimen, we may observe differences in MDM response to the viral insult. This study, following a subset of data presented in earlier publications on the transcriptomic profile response of MDMs isolated from healthy subjects and exposed to the active and the UV-inactivated SARS-CoV-2 VOCs, aims to elucidate the transcriptomic profiles of human MDMs isolated from four kidney transplant patients under immunosuppressive therapy in comparison to healthy human subjects.

## 2. Results

### 2.1. MDMs of Immunosuppressed Patients Revealed No Alterations in Their Transcriptomic Profiles in Response to SARS-CoV-2 Exposure

**Figure 1:**
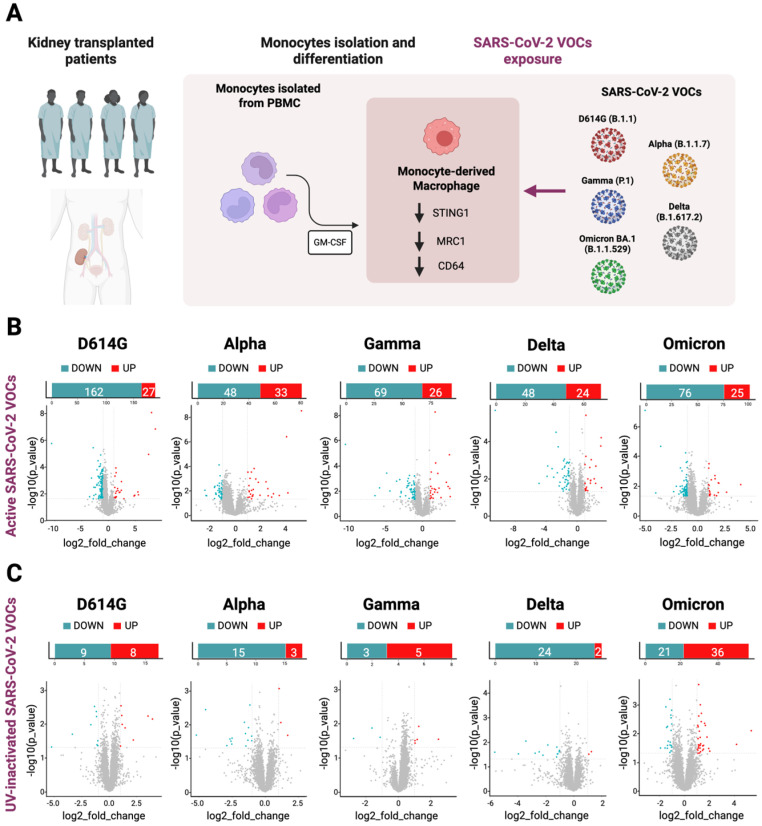
MDMs of immunosuppressed patients exposed to SARS-CoV-2 VOCs do not show a global alteration in their transcriptomic profiles in response to the virus. (**A**) General workflow used in this study to characterize macrophages’ response to SARS-CoV-2 VOCs exposure. PBMCs were collected from a group of four immunosuppressed kidney transplant patients and used to isolate monocytes subsequently differentiated with GM-CSF into macrophages and exposed to the active and the UV-inactivated SARS-CoV-2 VOCs (D614G, Alpha (B.1.1.7), Gamma (P1), Delta (B.1.617.2), and Omicron (BA.1)). RNA from these samples was then used to perform RNA-Seq experiments. (**B**) Volcano plots of RNA-Seq data showing the differentially expressed genes (DEGs, 1 < Log2_FC < −1, *p* < 0.05) in macrophages exposed to active D614G, Alpha, Delta, Gamma, and Omicron variants over the MDMs of immunosuppressed patients not exposed to SARS-CoV-2 VOCs used as control. Red dots represent up-regulated genes, and blue dots the down-regulated genes. (**C**) Volcano plots of RNA-Seq data showing the differentially expressed genes (DEGs, 1 < Log2_FC < −1, *p* < 0.05) in macrophages exposed to UV-inactivated D614G, Alpha, Delta, Gamma, and Omicron variants over each active counterpart. Red dots represent up-regulated genes and blue dots the down-regulated genes. This image was created using BioRender (https://www.biorender.com/).

Human monocytes were isolated from the PBMCs of four kidney transplant patients. After the differentiation into macrophages, those cells were exposed to the active and UV-inactivated particles of the different SARS-CoV-2 VOCs (D614G, Alpha B.1.1.7, Gamma P.1, Delta B.1.617.2 and Omicron BA.1) and then RNA-Seq analysis was performed (Figure 1A). The overall analysis of the transcriptomic data from the immunosuppressed MDMs exposed to the active SARS-CoV-2 VOCs did not show significant differences when compared to the not-exposed MDMs control group, except for the few protein-coding genes and non-coding RNAs shown in Supplementary File S1. In particular, in D614G-exposed MDM, 27 genes were significantly up-regulated and 161 were down-regulated. Alpha-exposed MDMs showed 33 significantly up-regulated genes and 48 down-regulated genes; in Gamma-exposed MDM, 26 genes were significantly up-regulated and 68 were down-regulated; Delta-exposed MDMs showed 24 significantly up-regulated genes and 84 down-regulated genes; and finally, 25 significantly up-regulated and 76 down-regulated genes were seen in the SARS-CoV-2 Omicron-exposed MDMs (Figure 1B).

Moreover, we compared the RNA-Seq results here obtained from MDMs exposed to the inactivated SARS-CoV-2 VOCs to each active counterpart. As shown in the volcano plots, we detected few significant differences in the transcriptomic profiles between the two datasets (Figure 1C). The complete list of genes is present in the Supplementary File S1.

Given that macrophages play a key role in pathogen recognition and immune activation, we hypothesized that the weak transcriptomic alterations shown by MDMs exposed to both active or UV-inactivated SARS-CoV-2 VOCs might be due to an impaired viral entry or replication within MDMs. To test this, we quantified SARS-CoV-2 transcripts by analyzing the expression of the major viral ORFs, including ORF1a, ORF1b, ORF3a, ORF6, ORF7a, ORF7b, ORF8, ORF10, S (spike protein), E (envelope), M (membrane), and N (nucleocapsid).

Surprisingly, we found that the main SARS-CoV-2 ORFs are highly transcribed in the MDM cells of immunosuppressed patients exposed to the viral particles, as shown in Table 1.

### 2.2. Integrated Analysis of RNA-Seq Data from Immunosuppressed and Immunocompetent Patients

In the attempt to better characterize transcriptional changes in macrophages isolated from patients under immunosuppressive therapies in response to viral infections, a comparison between immunosuppressed and immunocompetent patients is necessary. Hence, these transcriptomic results were compared to the subset of the data previously presented and published, obtained from immunocompetent participants [3]. In particular, monocytes isolated from PBMCs of immunocompetent participants were previously differentiated into macrophages and exposed to the SARS-CoV-2 strains D614G (the Italian Reference strain), Alpha (B.1.1.7), Delta (B.1.617.2), Gamma (P1), and Omicron (BA.1), as previously described [3].

**Figure 2:**
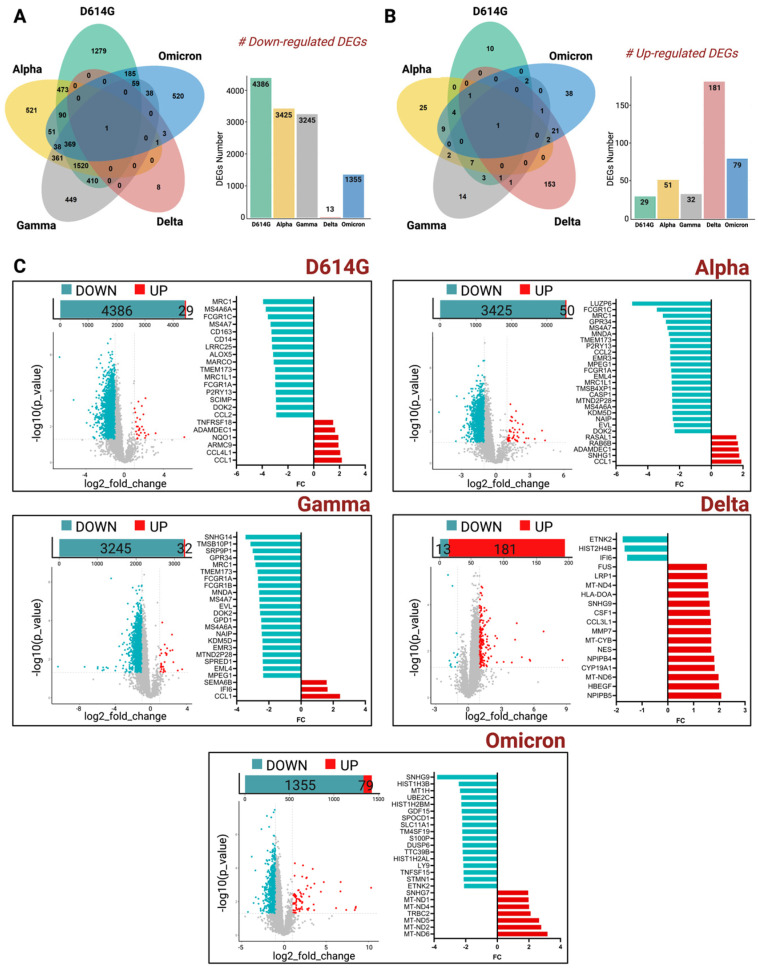
Comparison of MDMs of immunosuppressed patients exposed to SARS-CoV-2 VOCs and MDMs of immunocompetent subjects’ profile analysis. (**A**,**B**) Venn diagrams showing the total number of significantly down-regulated (**A**) and up-regulated (**B**) genes (*p*-value ≤ 0.05) across macrophages exposed to, D614G, Alpha, Delta, Gamma, and Omicron variants. Venn diagrams were generated using the R statistical software v4.3.2 (https://www.r-project.org/). (**C**) Left panels show volcano plots of RNA-Seq data showing the differentially expressed genes (DEGs, 1 < Log2_FC < −1, *p* < 0.05) in MDMs of immunosuppressed patients exposed to active D614G, Alpha, Delta, Gamma, and Omicron variants compared to MDMs of immunocompetent subjects. Red dots represent up-regulated genes, and blue dots the down-regulated genes; right panels show the top 20 (over present) significantly expressed genes (DEGs, 1 < Log2_FC < −1, *p* < 0.05) up and down regulated. Statistical significance of each sample was calculated over the control by using Student’s *t*-test. This image was created using BioRender (https://www.biorender.com/).

Strikingly, by performing this comparative analysis, thousands of genes were shown to be down-regulated across all the variants in the MDMs of immunosuppressed patients. Moreover, Venn diagrams showed that among all the genes that were down- and up-regulated, almost none of them were common to all the VOCs used in this study (Figure 2A,B). A high number of deregulated genes, instead, are shown to be common among D614G, Alpha, and Gamma variants. Indeed, considering the down-regulated DEGs, we detected 473 DEGs in common between D614G-exposed MDMs and Alpha-exposed MDMs, and 410 DEGs in common between D614G-exposed MDMs and Gamma-exposed MDMs (Figure 2A). Conversely, the SARS-CoV-2 Delta and Omicron-strain-exposed MDMs showed a lower number of common DEGs with D614G, Alpha, and Gamma VOCs. These findings correlate with the temporal evolution of SARS-CoV-2 virus, thus highlighting the genomic distance between the first strains and the most recent ones.

Stratifying all the transcriptomics data by each single VOC, the MDMs of immunosuppressed patients exposed to D614G, Alpha, and Gamma VOCs showed that thousands of genes were down-regulated when each of them was compared to the MDMs of immunocompetent participants’ data (Figure 2C).

The effect of the Delta variant on MDMs isolated from the two groups of patients, instead, did not show any difference in terms of down-regulated genes, as shown in the volcano plots in Figure 2C.

Interestingly, in a more detailed investigation of the list of genes that were found to be down-regulated in D614G, Alpha, and Gamma VOCs, we found important key players of innate immunity, like TMEM173 [11], and of macrophages polarization such as MRC1 and FCGR1A [12,13], as shown in the histogram bars of Figure 2C. The complete list of coding genes is presented in the Supplementary File S1.

The up-regulated DEGs, instead, showed an inverse pattern. Indeed, MDMs isolated from immunosuppressed patients and exposed to Delta, mainly, and Omicron VOCs, showed a slightly higher number of DEGs compared to the other VOCs, most of them involved in the mitochondrial metabolism of macrophages.

## 3. Discussion

The use of immunosuppressive drugs in clinics increased the preservation of organs’ functionality and decreased the percentage of rejections following transplantation [14]. It has been previously demonstrated that immunosuppressed subjects, such as transplanted patients, are less protected by vaccines compared to the general population and show a higher risk of prolonged infection with SARS-CoV-2 [10].

The immune system response to SARS-CoV-2 infection is characterized by the interplay between innate and adaptive immunity and, with this integrated system, immunocompetent individuals are generally able to accomplish viral clearance. Among the main players of the innate immune system, macrophages are considered crucial both for their role in antigen-presenting cells (APC) and in their function in regulate innate and adaptive immune responses. In this study, we wondered whether MDMs isolated from transplanted patients under an immunosuppressive therapeutic regimen may be altered in their response to the viral infection and this could be observed in their transcriptomic profiles when those cells were exposed to SARS-CoV-2 VOCs.

At first, by analyzing RNA-Seq data from MDMs exposed to all the SARS-CoV-2 VOCs and the control MDMs not exposed to the viral particles, we did not observe striking differences at the transcriptional level. Therefore, in order to exclude the possibility that in those cells the virus was not able to replicate at all, we did check the viral counts of the main ORFs of SARS-CoV-2 virus. The transcripts count analysis of the main SARS-CoV-2 ORFs did show that all the VOCs were highly replicating inside the MDM, thus suggesting that the weak alterations observed in the general transcriptomic profiles of MDMs isolated from the immunosuppressed patients may not be due to an ineffective infection but, most likely, to an alteration of the response to the viral insult, caused by the immunosuppressive therapy. Indeed, the group of patients comprising the object of this study was under a maintenance regimen of immune-suppressive drugs, provided after the kidney transplant, when the PBMCs were collected. In particular, the therapy administrated included tacrolimus, mycophenolate mofetil (MMF), and prednisone for three patients, while one patient was treated with tacrolimus, everolimus, and prednisone. Considering the well-established role of tacrolimus in impairing host innate immune responses [15] and in suppressing macrophages activation [16], and the impact of everolimus treatment in triggering cytokine release in macrophages [17], we may speculate that the effects observed in the transcriptomic profiles of these cells may be related to the immunosuppressive therapy.

Moreover, when comparing bulk RNA-Seq data from the MDMs of immunosuppressed patients and the data previously obtained from immunocompetent participants [3], we did observe a strong down-regulation of key genes of the innate immunity pathways. These alterations do not seem related to differences in the viral replication among the two groups of patients, considering the high number of viral counts of SARS-CoV-2 ORFs present in both groups of patients (Table 1). Indeed, the comparison of the viral counts of SARS-CoV-2 ORFs between immunosuppressed and immunocompetent patients showed no striking differences able to justify the transcriptional changes observed.

Interestingly, among the genes that were down-regulated in D614G, Alpha and Gamma VOCs, we found important key players of innate immunity, like TMEM173, also known as STING1, which plays a key role in the production of type I interferons (IFNs) and pro-inflammatory cytokines, as well as in autophagy and cell death mechanisms in response to pathogens infections [18]. The down-regulation of STING1 expression may have led to a weaker response from the host against the infections. MRC1 was also found to be down-regulated in the MDMs of immunosuppressed patients and this can also contribute to a less efficient innate host defense mechanism, considering its important role against viral infection. Given their central role in antiviral defense, the expression level of both TMEM173 and MRC1 could potentially serve as a useful biomarker to monitor innate immune competence in immunosuppressed transplant patients.

Moreover, the down-regulation of FCGR1A (CD64) also deserves special attention. Indeed, FCGR1A encodes a high-affinity receptor for the Fc portion of IgG, which is essential not only for antibody-dependent phagocytosis but also for antigen uptake, immune complex clearance, and subsequent antigen presentation to T cells [13]. Its reduced expression may therefore severely impair the capacity of macrophages to eliminate *opsonized* viral particles or infected cells, dampening the bridge between innate recognition and adaptive immune activation. Furthermore, Fcγ receptor-mediated signaling has been reported to influence cytokine production and the balance between pro-inflammatory and regulatory responses, meaning that the down-regulation of FCGR1A could contribute to both ineffective pathogen clearance and an altered inflammatory profile [19]. Considering the centrality of Fc receptor-mediated pathways in antiviral defense, the attenuated expression of FCGR1A in immunosuppressed patients suggests a profound functional impairment of macrophages that extends beyond canonical innate signaling and polarization programs. This impairment could ultimately compromise the efficiency of antibody-driven immunity, reduce the effectiveness of vaccination-induced responses, and exacerbate susceptibility to viral persistence or reactivation.

Although this study is limited due to the small sample size, which may limit the statistical relevance of the findings, with the data obtained by comparing immune-suppressed and immunocompetent patients, we could speculate that the different responses observed in the two groups of patients may be due to the inability of MDMs isolated from the transplanted immunosuppressed patients to act as main players in the first line of defense against the viral infections [20]. Furthermore, considering the role of macrophages as reservoirs of viruses [21], in these patients they could also be involved in a more aggressive infectious disease. The observed hypo-responsiveness of MDMs in immunosuppressed individuals may have important clinical implications. A dampened innate immune activation could contribute not only to delayed viral clearance but also to prolonged viral replication and persistence within host tissues. This condition may predispose transplant recipients to recurrent or chronic viral infections and potentially facilitate the emergence of immune escape variants.

## 4. Materials and Methods

### 4.1. SARS-CoV-2 Variants

SARS-CoV-2 isolation, propagation, titration, and infection were performed in a Biosafety Level 3 (BSL3) laboratory. All SARS-CoV-2 strains—Italian Reference D614G (B.1.1), Alpha (B.1.1.7), Gamma (P1), Delta (B.1.617.2), and Omicron BA.1 (B.1.1.529)—were isolated from nasopharyngeal swabs, and propagated onto a VERO C1008 (Vero 76, clone E6, Vero E6; ATCC CRL-1586TM) cell line. Genome sequencing was performed in order to confirm the presence of variant-defining mutations [22], and sequences were submitted to GISAID. The titration of virus variants and CPE were then performed, as previously described [3].

### 4.2. Cell Cultures and Macrophage Differentiation

Residual stored PBMCs from 4 kidney transplant patients, under maintenance immunosuppression (three of them with tacrolimus, mycophenolate mofetil and prednisone; 1 patient with tacrolimus, everolimus and prednisone), were plated in RPMI 1640 w/o FBS at a concentration of 5 × 10^6^ cells/well in 24-well microplates (COSTAR, Corning Incorporated, Corning, NY 14831, USA) for 3 h at 37 °C in a 5% CO_2_ atmosphere to let the cells adhere. Non-adherent cells were removed by gentle washing of the plate in PBS 1X three times; attached cells were then differentiated by stimulation with 800 U/mL Granulocyte-Macrophage Colony-Stimulating Factor (GM-CSF; Thermo Fisher, Waltham, MA 02451, USA) in RPMI 1640 w/10% *v*/*v* FBS for 7 days. Medium was changed once after 3 days at the same condition. The cells were gathered according to the Ethics Committee of IRCCS Policlinico San Matteo (P-0103662/21).

As a control group of patients, peripheral blood mononuclear cells (PBMCs) were isolated from eight buffy-coat units from healthy blood donors, cultured, and differentiated as previously described [3]. All the samples, from both the control group and kidney transplant patients, were processed at the same time and using the same protocols.

### 4.3. Macrophage Exposition to Viable and UV-Inactivated SARS-CoV-2 Variants

For the UV-inactivated SARS-CoV-2 experiments, 10 mL of 10,000TCID50/mL viable SARS-CoV-2 was exposed to direct UV-light for 2 h at room temperature, aliquoted, and stored at −80 °C. To assess viral inactivation, 200 µL was inoculated on VERO E6 (Vero 76, clone E6, Vero E6; ATCC CRL-1586TM) cells, and plates were checked daily. No cytopathic effect was detected up to 10 days post-inoculum. Macrophages were exposed to 200 µL of 10,000TCID50/mL of either viable or UV-inactivated SARS-CoV-2, for 2 h at 33 °C in a 5% CO_2_ atmosphere. After exposure, the inoculum was removed, and fresh RPMI 1640 w/o FBS was added. After 24 h of incubation, the supernatant was discarded, and cells were treated with 700 µL TRIzol (Thermo Fisher) and immediately stored at −80 °C until use for RNA extraction. At the same time, macrophages not exposed to SARS-CoV-2 were treated at the same conditions, minus exposure to SARS-CoV-2, and used as the naïve control group for all experiments.

### 4.4. RNA Extraction and Next-Generation Sequencing

Total RNA extraction was performed by using the RNeasy Mini kit (QIAGEN, Hilden, Germany), while RNA integrity was evaluated with the Agilent 4200 TapeStation System (Agilent Technologies Ltd., Santa Clara, CA, USA). RNA-Seq libraries were prepared using Illumina TruSeq Stranded Total RNA (Illumina, San Diego, CA, USA) and TruSeq RNA UD Indexes 24 Indexes-96 Samples (Illumina), as previously described [4]. Sequencing was performed on a NextSeq™ 550 (Illumina) with 2 × 76 cycles, following the manufacturer’s instructions.

### 4.5. Bioinformatic Analysis of RNA-Seq Data

Bioinformatic analysis of the RNA-Seq data was performed as previously described [3]. Briefly, raw data files in FASTQ generated from the NextSeq550 system (Illumina) were checked for quality control and low-quality reads filtering and adapter trimming were performed. Processed reads were then aligned to the human reference genome (hg19) with STAR (v2.7.0) (https://github.com/alexdobin/STAR/releases) [23]. Quantification of the transcript abundances was performed with RSEM (v1.3.3) (https://deweylab.github.io/RSEM/) [24], and expression levels were normalized for kilobase of transcript per million (TPM). All the transcripts that were below the threshold of 10 normalized read counts, after the averaging across all the biological replicates, were excluded from the analysis. Differential expression analysis was performed as previously described [3]. Briefly, gene expression values were modified by applying a base-2 logarithm to the normalized TPM values plus 1, to avoid the logarithm of zero, i.e., log2(TPM + 1). Then, in order to identify differentially expressed genes (DEGs), Student’s *t*-test was performed, with a significance threshold set at a *p*-value of <0.05. The test was applied for each pair of conditions only when at least 3 out of 4 samples exhibited log2(TPM + 1) > 1, ensuring that genes with low expression across most samples were excluded from the analysis in order to minimize the likelihood of false discoveries

R (v4.3.2) and Python (v3.8.10) were used for data analysis. Volcano plots were used to visualize down- and up-regulated DEGs for each variant. Venn diagrams were generated using the R statistical software v4.3.2 (https://www.r-project.org/).

## 5. Conclusions

Although further studies and larger cohorts of patients are required to fully elucidate these observations, our data pave the way for new studies and may provide insights for a deeper characterization of the response of macrophages to viral stimuli, particularly in immunosuppressed patients. Indeed, while regulatory cells, especially T cells, have been extensively studied and characterized, considering their pivotal role in the host defense [25], very little is known about the role of macrophages in this particular scenario. Understanding these mechanisms could provide valuable insights into the therapeutic strategies required to enhance antiviral immunity in immunosuppressed patients.

## Figures and Tables

**Table 1:** SARS-CoV-2 VOCs ORFs transcripts counts.

Viable Viral Particles												
	Immuno-Competent						Immuno-Suppressed					
	CTRL	D614G	Alpha	Delta	Gamma	Omicron	CTRL	D614G	Alpha	Delta	Gamma	Omicron
ORF1a	0	7895	300	307,054	4940	179,915	0	1806	3295	21,063	33,850	10,642
ORF1b	0	7	15	4	5	6	0	5	6	5	6	5
S	0	1420	52	49,558	888	31,109	0	320	581	3803	5753	1699
N	0	698	29	15,510	468	14,194	0	123	219	2537	2463	585
ORF3a	2	243	10	7879	159	5391	0	41	83	636	921	224
M	0	257	11	7872	168	5381	0	58	92	727	942	312
ORF8	0	114	4	2947	64	1987	0	26	40	319	419	116
ORF7a	0	88	8	2090	72	1727	0	16	28	220	328	68
E	0	31	2	1034	18	578	0	6	15	74	105	35
ORF6	0	23	1	520	6	320	0	7	5	41	39	30
ORF7b	0	2	0	39	3	44	0	0	1	3	3	3
ORF10	0	7	0	52	4	93	0	1	0	12	11	3

MDMs isolated from both immune-competent and immune-suppressed patients were exposed to viable SARS-CoV-2 viral particles and RNA-Seq was performed. On the left, in capital letters, there is the list of the 12 ORFs checked to assess the viral replication. Control groups, indicated as CTRL in the table, represent the same MDM cells not exposed to the virus.

## Data Availability

The datasets used and analyzed during the current study are available from the corresponding author upon reasonable request.

## Supplementary Materials

The following supporting information can be downloaded at https://www.mdpi.com/article/10.3390/ijms262110489/s1.

## Abbreviations

The following abbreviations are used in this manuscript:

SARS-CoV-2	Severe Acute Respiratory Syndrome Coronavirus 2
VOC	Variants of Concern
MDM	Monocytes Derived Macrophages
TPM	Transcripts per Million
